# Pre-habilitation Before Vestibular Schwannoma Surgery—Impact of Intratympanal Gentamicin Application on the Vestibulo-Ocular Reflex

**DOI:** 10.3389/fneur.2021.633356

**Published:** 2021-02-09

**Authors:** Alexander A. Tarnutzer, Christopher J. Bockisch, Elena Buffone, Alexander M. Huber, Vincent G. Wettstein, Konrad P. Weber

**Affiliations:** ^1^Department of Neurology, University Hospital Zurich, Zurich, Switzerland; ^2^Faculty of Medicine, University of Zurich, Zurich, Switzerland; ^3^Neurology, Cantonal Hospital of Baden, Baden, Switzerland; ^4^Clinical Neuroscience Center, Zurich, Switzerland; ^5^Department of Otorhinolaryngology, University Hospital Zurich, Zurich, Switzerland; ^6^Department of Ophthalmology, University Hospital Zurich, Zurich, Switzerland; ^7^Rautipraxis AG, Zurich, Switzerland

**Keywords:** video-head-impulse testing, aminoglycosides, vestibulotoxicity, tumor size, anterior-canal sparing

## Abstract

**Background:** Patients with vestibular schwannoma that show residual peripheral-vestibular function before surgery may experience sudden and substantial vestibular loss of function after surgical resection. To alleviate the sudden loss of peripheral-vestibular function after vestibular-schwannoma (VS) resection, pre-surgical intratympanic gentamicin application was proposed.

**Objective:** We hypothesized that this approach allows for a controlled reduction of peripheral-vestibular function before surgery but that resulting peripheral-vestibular deficits may be canal-specific with anterior-canal sparing as observed previously in systemic gentamicin application.

**Methods:** Thirty-four patients (age-range = 27–70 y) with unilateral VS (size = 2–50 mm) were included in this retrospective single-center trial. The angular vestibulo-ocular reflex (aVOR) was quantified before and after (29.7 ± 18.7 d, mean ± 1SD) a single or two sequential intratympanic gentamicin applications by use of video-head-impulse testing. Both aVOR gains, cumulative saccadic amplitudes, and overall aVOR function were retrieved. Statistical analysis was done using a generalized linear model.

**Results:** At baseline, loss of function of the horizontal (20/34) and posterior (21/34) canal was significantly (*p* < 0.001) more frequent than that of the anterior canal (5/34). After gentamicin application, loss of function of the horizontal (32/34) or posterior (31/34) canal remained significantly (*p* ≤ 0.003) more frequent than that of the anterior canal (18/34). For all ipsilesional canals, significant aVOR-gain reductions and cumulative-saccadic-amplitude increases were noted after gentamicin. For the horizontal canal, loss of function was significantly larger (increase in cumulative-saccadic-amplitude: 1.6 ± 2.0 vs. 0.8 ± 1.2, *p* = 0.007) or showed a trend to larger changes (decrease in aVOR-gain: 0.24 ± 0.22 vs. 0.13 ± 0.29, *p* = 0.069) than for the anterior canal.

**Conclusions:** Intratympanic gentamicin application resulted in a substantial reduction in peripheral-vestibular function in all three ipsilesional canals. Relative sparing of anterior-canal function noted at baseline was preserved after gentamicin treatment. Thus, pre-surgical intratympanic gentamicin is a suitable preparatory procedure for reducing the drop in peripheral-vestibular function after VS-resection. The reasons for relative sparing of the anterior canal remain unclear.

## Introduction

Treatment options for patients with growing vestibular schwannoma (VS) or with local compressive effects include radiosurgery and microsurgical resection (1–4). As a potential side effect of treatment, those patients with residual peripheral-vestibular function may experience sudden and substantial vestibular loss of function (5). To reduce such side effects due to dissection of the vestibular nerve, drug-induced ablation of ipsilateral peripheral-vestibular function by use of vestibulotoxic substances has been proposed as a pre-surgical treatment (6–9). Specifically, there is a level-3 recommendation from the Congress of Neurological Surgeons on pre-operative gentamicin ablation intratympanically to induce a controlled partial loss of semicircular canal (SCC) function and to improve post-operative mobility (10). Thus, after surgical VS resection, the delta in loss of function is presumably smaller than in untreated patients and their clinical symptoms after surgery will be smaller (11). This may have a positive impact on rehabilitation and recovery (12), including coping with vertigo and (multi)sensory input (9, 13–15). Furthermore, the gentamicin-induced pre-surgical loss of vestibular function will occur while the patient is in his/her natural state of health and mobility, which may facilitate recovery compared to his/her condition immediately after surgery.

Aminoglycosides are known for causing vestibular loss of function when applied intravenously (16–19), albeit hearing may deteriorate also (20). Recovery is usually limited, and underlying pathomechanisms of aminoglycoside-induced vestibulotoxicity are still unclear (21). Previously, relative sparing of the anterior canal(s) after aminoglycoside treatment has been reported (22). Whether this is true also for patients who received intratympanic aminoglycosides as part of pre-surgical treatment is not known. As intratympanic gentamicin application is an established, efficient treatment for Menière's disease (MD) (23, 24), this approach may serve as a model to study the vulnerability of the SCCs to pre-defined doses of gentamicin. Thus, we hypothesized that relative sparing of anterior-canal function will be present also in patients who received intratympanic gentamicin before VS resection. Alternatively, with a comparable reduction in SCC function for all three canals, this would speak against a selective vulnerability of specific SSCs to intratympanic aminoglycosides.

To test this hypothesis, we quantified SCC function before and after intratympanic gentamicin application in VS patients and compared the loss of function in individual canals. We predicted a significantly smaller loss of function in the ipsilesional anterior canal compared to the posterior and horizontal canal.

## Materials and Methods

The local ethics committee (Cantonal Ethics Committee Zurich) approved the experimental protocol. The protocol was in accordance with the ethical standards of the 2013 Declaration of Helsinki for research involving human beings. All subjects that had been treated after January 1st 2016 had previously provided written general consent for the use of health-related data and samples for research purposes, whereas those treated earlier could be included based on the approval of the study protocol by the local Ethics committee (study protocol 2018-00224). We retrospectively screened the Hospital's electronic files for patients who have received a diagnosis of unilateral VS and treatment with gentamicin prior to surgical resection between May 2013 and September 2017. Ten patients from the current study were previously published (25).

### Intratympanic Gentamicin Treatment

All patients received a treatment with intratympanic gentamicin (solution = 80 mg/2 ml) ipsilaterally to the VS applied by an ENT specialist at least 6 weeks before surgery. vHIT was obtained at baseline and 2–6 weeks after gentamicin treatment. If loss of function was found to be insufficient, a second gentamicin dose was administered. Dosage of gentamicin ranged between 0.25 and 1 ml, depending on the volume of the tympanic cavity. Pure tone audiometry (PTA) was obtained in all patients at baseline and after treatment.

### vHIT-Recording Procedure

All patients received quantitative vestibular testing before and after intratympanic gentamicin application. We required 20 valid head impulses for each canal (26), with SCCs tested in pairs according to the planes of stimulation (horizontal canals, RALP plane for right anterior and left posterior canal, LARP plane for left anterior and right posterior canal). For video-oculography, we used commercially available vHIT goggles (Otometrics, Taastrup, Denmark) with an infrared camera recording the right eye. Horizontal and vertical eye position was measured (frequency = 250 Hz), and angular head velocity was determined by three orthogonal mini-gyroscopes. For further analysis, eye-velocity values were calculated.

### Patient Identification and Data Analysis

We reanalyzed angular VOR (aVOR) gains in all patients using Otosuite 4.0 (Otometrics, Taastrup, Denmark) and ran custom-written MATLAB (R2017b, The MathWorks, Natick, MA, USA) routines for the quantification of overt corrective saccades, calculating cumulative saccadic amplitudes (CSA) per trial (22). For this study, we read out the standard aVOR gain calculations from the Otometrics vHIT goggles. Their algorithm calculates gain as the ratio of the area under the desaccaded eye-velocity curve to the area under the head-velocity curve, corresponding to a desaccaded position gain (27). Thus, the gain of the aVOR was calculated as the ratio of cumulative slow-phase eye velocity over cumulative head velocity from the onset of the head impulse to the moment when head velocity crossed zero again (27). As the amplitude of covert saccades on top of the (residual) aVOR response is ill-defined and hard to calculate reliably, we chose to only include overt saccades for our analysis (17, 28). Saccades were defined as “overt,” if their onset occurred after head velocity crossed zero after the head impulse. Vestibular hypofunction was defined as a reduction in aVOR gain and/or the occurrence of compensatory saccades. For a diagnosis of unilateral-vestibular loss (UVL), hypofunction of at least one canal on one side was required.

For gains, cutoff values of 0.8 (horizontal canals) and 0.7 (vertical canals) have been proposed by the manufacturer (Otometrics) to distinguish normal from reduced aVOR function. Previously proposed cutoff values suggested that the CSA > 0.7–0.8°/trial indicates loss of function of the canal tested (22, 29). Here we adhered to the cutoff value (0.73°/trial) proposed by our group (22), as the same statistical approach was used.

On MR imaging (obtained in all patients), the maximal diameter of the tumor was determined. Two experienced neuro-ontologists (KPW, AAT) independently reviewed all vHIT traces. Traces were evaluated for reduced aVOR gain, increased CSA, or a combination of both (22). Inter-rater agreement for individual canal function (normal vs. pathological) was 0.83 (Cohen's kappa) (30). Discordant ratings were resolved by discussion among the reviewers.

Individual patterns of SCC hypofunction were assessed. MATLAB and SPSS 24 (IBM, Armonk, NY, USA) were used for statistical analyses. Fisher's exact-test with Bonferroni correction for multiple tests was applied to determine significant differences in the occurrence of specific conditions. The level of significance for all statistical tests was 0.05. We applied a generalized linear model (GLM, SPSS 24) to analyze the effects of the gentamicin treatment on the extent of peripheral-vestibular impairment. Fisher's least significant difference (LSD) method was used to correct for multiple tests when performing pairwise comparisons.

Principal component analysis (PCA) was applied for comparisons between two dependent variables (31). The coefficient of determination (R^2^) was used to assess the goodness of fit. A correlation between two variables was considered significant whenever the 95% confidence interval (95% CI) of the slope did not include zero.

Standardized evaluation of hearing function on PTA was performed according to the CPT-AMA guidelines (32), assessing hearing at four different frequencies (500 Hz/1 kHz/2 kHz/4 kHz). Significant hearing loss was defined as a CPT value >20% on the affected side.

## Results

Between May 2013 and September 2017, 41 patients with unilateral VS received intratympanic gentamicin injections prior to VS resection at the University Hospital Zurich. From those subjects, seven were excluded due to missing/denied general consent (n=2) or due to missing post-gentamicin vHIT (*n* = 5). From the remaining 34 patients (aged = 27–70 years, 10 females), 26 received a single intratympanic gentamicin treatment, whereas eight patients received two doses. VS size varied between 2 and 50 mm (Table 1).

**Table 1:** Epidemiology—key facts.

Gender (*n*)		%
Females	10	29
Males	24	71
**Age (mean ± 1SD) (years)**		
Females	52.1 ± 11.9	
Males	50.5 ± 8.4	
**Affected side (*n*)**		
Right	14	41
Left*	20	59
**VS size (mm)**		
1–10	5	15
11–20	12	35
21–30	14	41
31–40	2	6
>40	1	3
Range	2–50	
Mean ± 1SD	21.4 ± 9.3	
**Gentamicin treatment sessions (*n*)**		
One session	26	76
Two sessions	8	24
**Gentamicin treatment dose (mean ± 1SD) (ml)**		
First session	0.50 ± 0.20^†^	
Second session	0.55 ± 0.14^‡^	
**Timing of vHIT testing relative to gentamicin treatment (days)**		
Delay gentamicin treatment—baseline vHIT (mean ± 1SD)	20.2 ± 21.1	
Delay FU vHIT testing—gentamicin treatment (mean ± 1SD)	29.7 ± 18.7	
Delay FU vHIT testing−2nd gentamicin treatment (mean ± 1SD)	26.9 ± 16.6	
Delay gentamicin treatment—surgery (mean ± 1SD)	57 ± 44	

*FU, follow-up*.

* *Results from patients with left-sided unilateral vestibular disease were mirrored for further analysis, so that the affected side is always on the right in the paper*.

† *Missing values from 3 patients*.

‡ *Missing values from 2 patients*.

**Figure 1:**
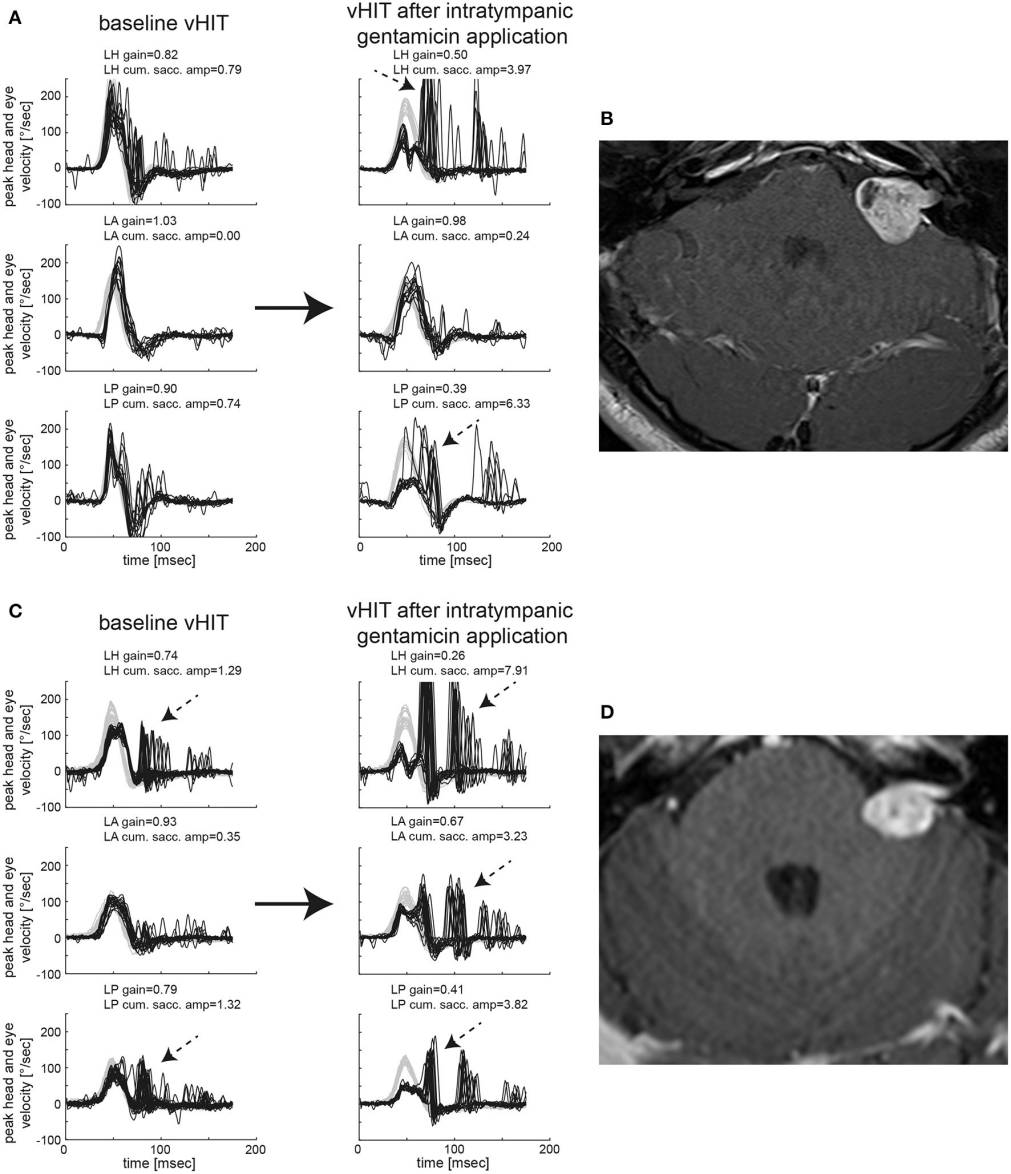
Video-head-impulse testing before and after intratympanic gentamicin application is shown for two representative patients with unilateral VS. In **(A)** [patient #14, left-sided VS, max. diameter 20 mm as shown on axial T1-weighted contrast-enhanced MRI in **(B)**], baseline testing showed an overall normal aVOR response for all six SCCs with only very few saccades for the ipsilesional horizontal and posterior canal (only ipsilesional traces shown). One month after intratympanic gentamicin application (0.3 ml), vHIT demonstrates a significantly reduced aVOR gain for the left horizontal and left posterior SCC with accompanying overt catch-up saccades, whereas the left anterior canal remained functionally intact. In addition, low-amplitude, compensatory saccades are observed in the right horizontal and posterior SCC. In **(C)** [patient #32, left-sided VS, max. diameter 19 mm as shown on axial T1-weighted contrast-enhanced MRI in **(D)**], baseline testing indicated partial loss of function of the ipsilesional horizontal (with mild reduction in gain and overt catch-up saccades) and posterior (with normal gain but significant overt catch-up saccades) SCC, whereas anterior-canal function remained intact. Sixteen days after left-sided intratympanic gentamicin application (0.7 ml), gains of the ipsilesional horizontal and posterior canal had dropped sharply with covert and overt catch-up saccades having grown in amplitude as shown on vHIT. In addition, the ipsilesional (left) anterior SCC now demonstrated a partial loss of function with a mild decrease in gain and significant covert and overt catch-up saccades. However, in comparison to the left horizontal and posterior canal, the impairment in aVOR was clearly smaller for the left anterior canal, suggesting gentamicin-related anterior-canal sparing.

In Figure 1, vHIT results and MR imaging from two subjects are shown, illustrating different patterns before and after gentamicin treatment. While the first subject (#14, panels AB) demonstrated normal SCC function ipsilesionally and relative sparing of anterior-canal function after gentamicin treatment, the second subject (#32, panels CD) presented with impaired horizontal/posterior canal function before treatment and loss of function of all three SCCs after gentamicin treatment. Residual gains, however, were highest for the anterior SCC, pointing to relative sparing of anterior-canal function.

### Video-Head-Impulse Testing in Vestibular Schwannoma–Baseline Testing

**Figure 2:**
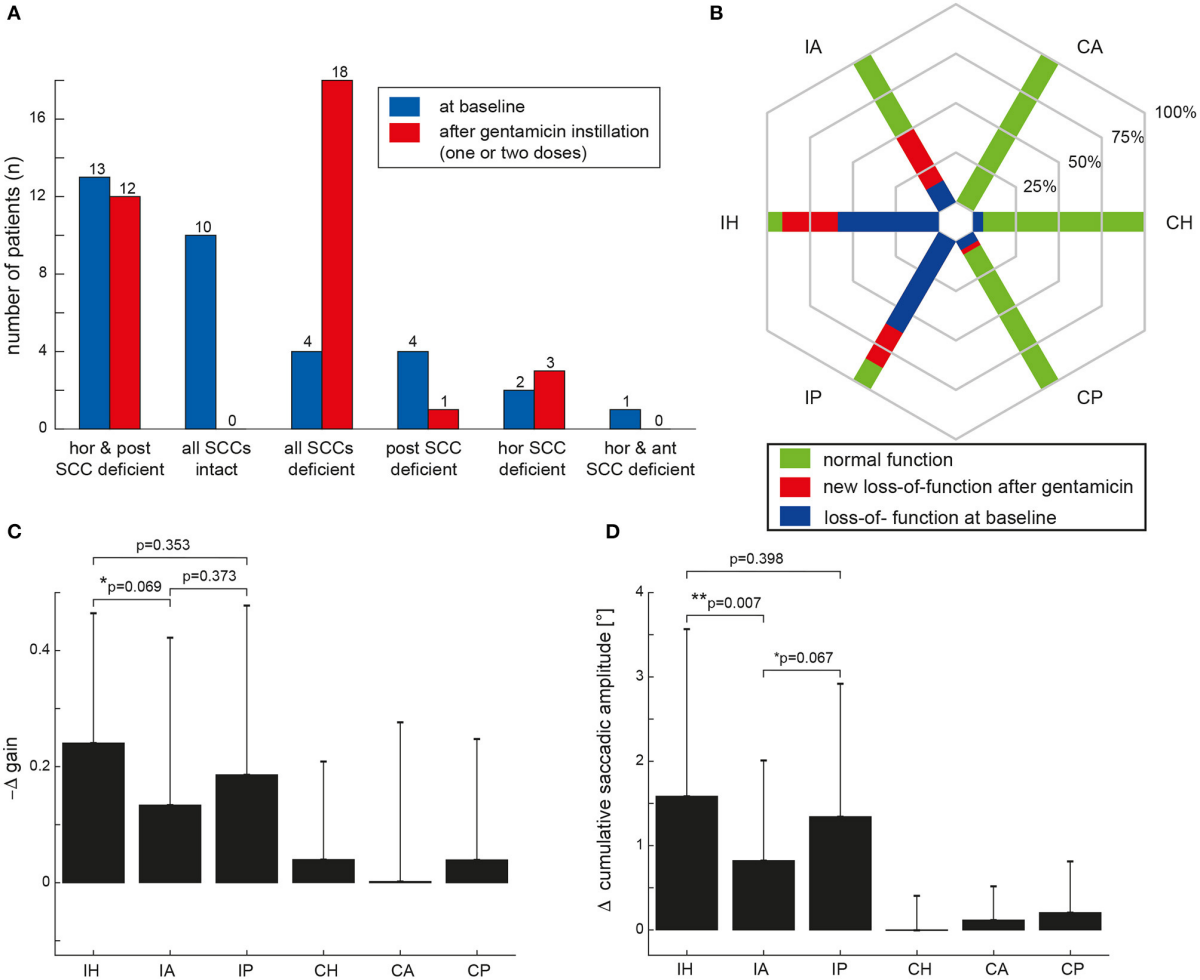
**(A)** Distribution of ipsilesional impairment in semicircular canal (SCC) function based on the two reviewers' ratings both at baseline (in blue) and after intratympanic gentamicin treatment (one or two treatments, individually different) (in red). In **(B)**, the percentage of patients with normal function before and after gentamicin instillation (green areas) and with hypofunction based on the reviewers' overall ratings for the different SCCs [horizontal (H), anterior (A) and posterior (P)] are illustrated in a hexplot, differentiating between the ipsilesional (I) and the contralesional (C) side in the plot. Loss of function at baseline (indicated by the blue bars) is distinguished from new SCC hypofunction after intratympanic gentamicin application (indicated by the red bars); thus, the overall fraction of SCC impairment after gentamicin treatment is reflected by the red plus the blue fraction. In **(C,D)**, the mean (±1 SD) changes (Δ) in aVOR gain **(C)** and CSA **(D)** after intratympanic gentamicin application compared to baseline measurements (i.e., by subtracting the aVOR gain/the CSA at baseline from the aVOR gain/CSA after gentamicin instillation) are illustrated for all six SCCs. Note that aVOR-gain reductions after gentamicin instillation will result in a negative Δ gain [as indicated along the y-axis in **(C)**]. Results of the statistical analysis (GLM) are shown for the ipsilesional (I) side only as there were no significant changes (*p* > 0.05) on the contralesional (C) side. Differences with a trend to significance were indicated by “*,” whereas significant (i.e., *p* < 0.05) differences were marked with a “**.”

At baseline, an ipsilesionally impaired aVOR was noted by the two reviewers in at least one SCC in 24/34 VS patients (71%), whereas SCC function remained intact in 10 patients (29%). Different distribution patterns of ipsilesional SCC function at baseline were observed (Figure 2A), with impaired horizontal/posterior canal function (38%), preserved peripheral-vestibular function in all three SCCs (29%), impaired SCC function in all three SCCs (12%), and impaired posterior-canal function (12%) being most frequent. The fraction of loss of function for the different ipsilesional SCCs is illustrated in Figure 2B. In comparison to impairment of the anterior SCC, loss of function of the horizontal (5 vs. 20; *p* < 0.001, Fisher's exact-test, Bonferroni corrected) or posterior (5 vs. 21; *p* < 0.001) SCC was significantly more frequent.

Statistical analysis (GLM) of vHIT gains at baseline and after gentamicin treatment showed a significant main effect for the condition (df = 1, chi-square = 29.322, *p* < 0.001) and individual ipsilesional SCCs (df = 2, chi-square = 134.373, *p* < 0.001). Furthermore, a significant interaction between these two parameters was noted (df = 5, chi-square = 19.261, *p* = 0.002). Likewise, statistical analysis of CSA demonstrated a significant main effect for the condition (df = 1, chi-square = 53.974, *p* < 0.001) and the SCCs (df = 2, chi-square = 284.151, *p* < 0.001). Again, a significant interaction was noted (df = 5, chi-square = 44.926, *p* < 0.001).

**Figure 3:**
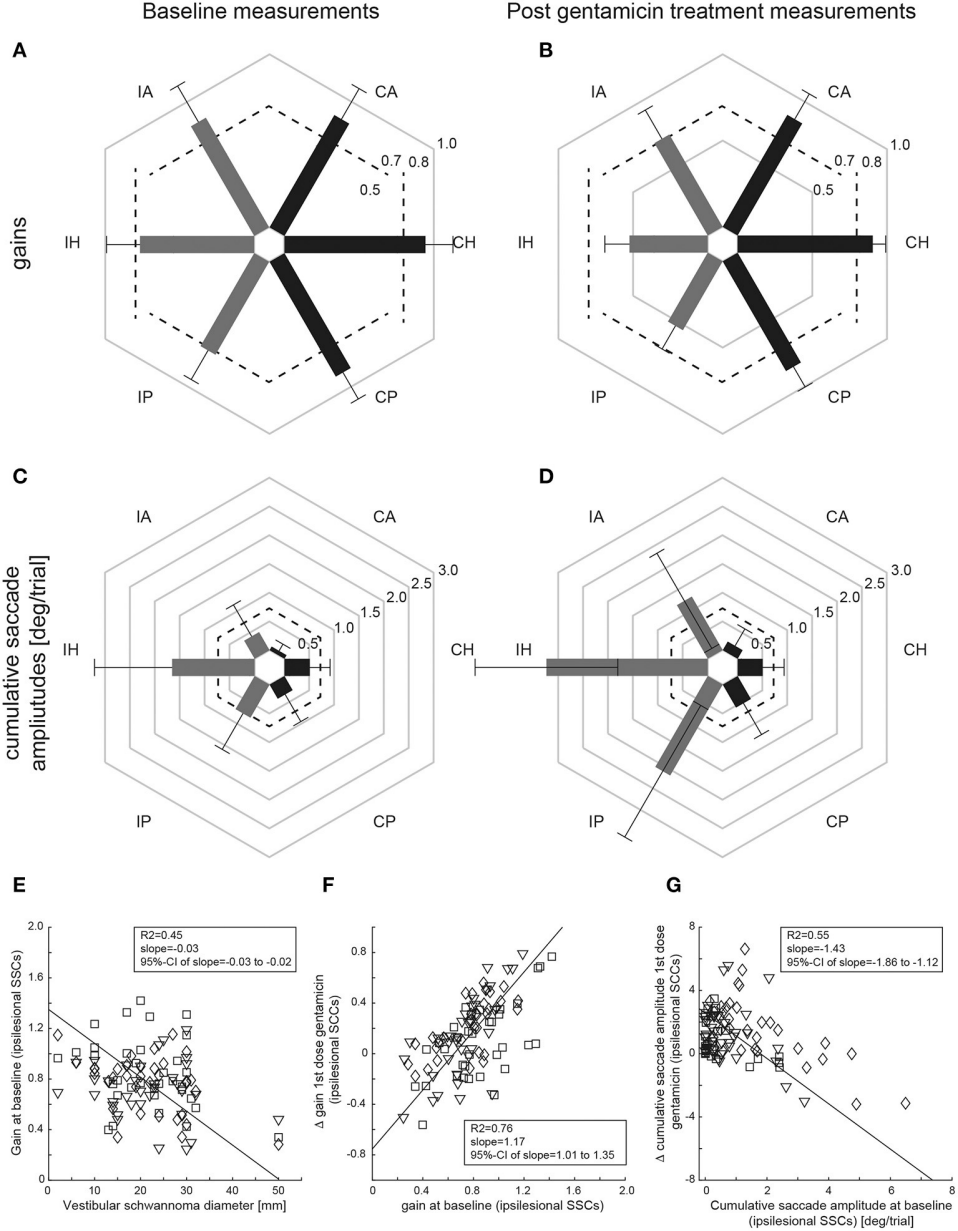
Mean (±1SD) gains **(A,C)** and CSA **(B,D)** of all patients (*n* = 34) are shown separately for baseline (left column) and after gentamicin treatment (right column), with values from the affected [ipsilesional (I)] side and the unaffected [contralesional (C)] side indicated by gray and dark bars, respectively. Gain values (from 0 to 1) and CSA (°/trial, from 0 to 3) are provided along the different hexagons. Cutoff values for reduced gains (<0.8 for the horizontal canals, <0.7 for the vertical canals) and for abnormally increased cumulative saccade amplitudes (>0.73°/trial) are indicated by dashed lines. In **(E–G)**, correlation analyses using principal component analysis (PCA) are shown both for the size of the VS and for the aVOR gain of the individual SSCs on the affected side at baseline **(E)**, the difference (Δ) in aVOR gain (gain after 1st dose of gentamicin subtracted from gain at baseline) vs. baseline gain **(F)** and for the difference (Δ) in CSA (CSA at baseline subtracted from CSA after 1st dose of gentamicin) vs. baseline cumulative saccade amplitude **(G)**. The diamonds (horizontal canals), squares (anterior canals), and inverted triangles (posterior canals) refer to single-subject and single SCC measurements; the solid black line represents the fit of the PCA, with details shown in the inlets [including the 95% confidence interval (CI) of the slope]. Note that individual results from the three ipsilesional SCCs are combined for the PCS.

Performing pairwise comparisons, ipsilesional mean gains (±1SD) at baseline (panel A) were significantly (*p* < 0.001) smaller than on the healthy side for the horizontal and posterior canal, but not for the anterior canal (*p* = 0.670) (Figure 3 and Table 2). Likewise, CSA at baseline (panel C) were significantly larger on the affected side than on the healthy side for the horizontal canal (*p* < 0.001) and showed a trend toward significance for the posterior canal (*p* = 0.052), whereas this was not the case for the anterior canal (*p* = 0.607).

**Table 2:** Ipsilesional aVOR-gains and CSA—statistical analysis.

	**Baseline aVOR gains**			**Post gentamicin aVOR gains**			**Baseline vs. post-gentamicin aVOR gains**
	**Ipsilesional**	**Contralesional**	**Statistical analysis ipsilesional vs. contralesional**	**Ipsilesional**	**Contralesional**	**Statistical analysis ipsilesional vs. contralesional**	**Statistical analysis ipsilesional SCCs**
Horizontal SCC	0.76 ± 0.22	0.94 ± 0.19	*p* < 0.001	0.52 ± 0.16	0.91 ± 0.09	*p* < 0.001	0.76 ± 0.22 vs. 0.52 ± 0.16, *p* < 0.001
Anterior SCC	0.85 ± 0.28	0.87 ± 0.24	*p* = 0.670	0.71 ± 0.22	0.87 ± 0.19	*p* = 0.002	0.85 ± 0.28 vs. 0.71 ± 0.22, *p* = 0.006
Posterior SCC	0.72 ± 0.23	0.89 ± 0.21	*p* < 0.001	0.53 ± 0.18	0.85 ± 0.16	*p* < 0.001	0.72 ± 0.23 vs. 0.53 ± 0.18, *p* < 0.001
	**Baseline CSA [°/trial]**			**Post gentamicin CSA [°/trial]**			**Baseline vs. post-gentamicin CSA [°/trial]**
	**Ipsilesional**	**Contralesional**	**Statistical analysis ipsilesional vs. contralesional**	**Ipsilesional**	**Contralesional**	**Statistical analysis ipsilesional vs. contralesional**	**Statistical analysis ipsilesional SCCs**
Horizontal SCC	1.66 ± 1.56	0.51 ± 0.41	*p* < 0.001	3.24 ± 1.43	0.50 ± 0.43	*p* < 0.001	1.66 ± 1.56 vs. 3.24 ± 1.43, *p* < 0.001
Anterior SCC	0.42 ± 0.73	0.08 ± 0.16	*p* = 0.607	1.24 ± 1.10	0.20 ± 0.39	*p* < 0.001	0.42 ± 0.73 vs. 1.24 ± 1.10, *p* < 0.001
Posterior SCC	0.77 ± 0.83	0.32 ± 0.63	*p* = 0.052	2.11 ± 1.53	0.53 ± 0.73	*p* < 0.001	0.77 ± 0.83 vs. 2.11 ± 1.53, *p* < 0.001

*aVOR, angular vestibulo-ocular reflex; CSA, cumulative saccadic amplitude; SCC, semicircular canal*.

In a next step, we asked whether VS size had an impact on the extent of peripheral-vestibular loss of function. Using PCA, individual vHIT gains at baseline from all three ipsilesional SCCs were compared with the VS diameter (Figure 3E), showing a significant inverse correlation [R^2^ = 0.45, slope = −0.03 (95%-CI = −0.03 to −0.02)].

### Video-Head-Impulse Testing After Intratympanic Gentamicin Injection

After gentamicin treatment, overall SCC function was impaired ipsilesionally in at least one SCC in all 34 patients (Figure 2A). Most often, impairment of all three SCCs was observed (53%), followed by loss of function restricted to the horizontal and posterior canal (35%) or the horizontal canal (9%).

The fraction of deficient canals grew after gentamicin treatment (Figure 2B). In comparison to impairment of the anterior SCC, loss of function of the horizontal (18 vs. 32; *p* < 0.001) or posterior (18 vs. 31; *p* = 0.003) SCC remained significantly more frequent after gentamicin treatment.

Performing pairwise comparisons, ipsilesional mean gains after gentamicin treatment (Figure 3B and Table 2) were significantly (*p* ≤ 0.002) smaller than on the contralesional side for all three SCCs. Likewise, CSA (Figure 3D) were significantly (*p* < 0.001) larger on the affected side than on the healthy side for all three SCCs.

### Changes in aVOR Gains and CSA After Gentamicin Treatment

On the affected side, aVOR gains were significantly (*p* ≤ 0.006) reduced and CSA were significantly (*p* < 0.001) increased for all three SCCs after gentamicin treatment. In contrast, no significant (*p* > 0.05) changes in vHIT gains and CSA after gentamicin treatment were noted contralesionally.

When comparing the delta (Δ) in aVOR gain for all SCCs, a significant main effect was noted (df = 5, chi-square = 25.860, *p* < 0.001) (Figure 2C). Pairwise comparisons demonstrated a trend toward a larger ipsilesional decrease in aVOR gain for the horizontal canal compared to the anterior canal after gentamicin treatment (0.24 ± 0.22 vs. 0.13 ± 0.29, *p* = 0.069), whereas no significant differences in ipsilesional ΔaVOR gain were noted when comparing the horizontal and posterior canal (0.24 ± 0.22 vs. 0.19 ± 0.29, *p* = 0.353) and the posterior and anterior canal (0.19 ± 0.29 vs. 0.13 ± 0.29, *p* = 0.373). When comparing the ΔCSA for all SCCs, a significant main effect was noted as well (df = 5, chi-square = 56.469, *p* < 0.001) (Figure 2D). The increase in CSA after gentamicin treatment was significantly larger for the horizontal canal than for the anterior canal (1.6 ± 2.0 vs. 0.8 ± 1.2, *p* = 0.007) and showed a trend toward a significantly larger increase for the posterior canal compared to the anterior canal (1.3 ± 1.6 vs. 0.8 ± 1.2, *p* = 0.067). No significant differences in ΔCSA were noted when comparing the horizontal and posterior ipsilesional canal (*p* = 0.398).

ΔaVOR gain (all ipsilesional SCCs pooled) after a first dose of gentamicin was compared with the aVOR gain at baseline, demonstrating a significant correlation [R^2^ = 0.76, slope = 1.17 (95%-CI = 1.01–1.35)] (Figure 3F). Likewise, ΔCSA after a first dose of gentamicin was inversely correlated with the CSA at baseline [R^2^ = 0.55, slope = −1.43 (95%-CI = −1.86 to −1.12)] (Figure 3G). In contrast, there was no correlation between the first gentamicin dose and the ΔaVOR-gain [R^2^ = 0.07, slope = −1.45 (95%-CI = −2.08 to 1.43)] or the ΔCSA [R^2^ = 0.08, slope = −8.66 (95%-CI = −12.13 to 8.30)].

### Hearing-Impairment at Baseline and After Gentamicin Treatment

Pure tone audiometry at baseline demonstrated significant hearing loss in 31/34 patients (91%) at 0.5 Hz (mean = 43 dB HL, range = 5–120), 1 Hz (mean = 58 dB HL, range = 10–120), 2 Hz (mean = 68 dB HL, range = 5–120), and 4 Hz (mean = 73 dB HL, range = 25–120), with an average CPT-AMA hearing loss (±1SD) of 64 ± 27%. After gentamicin treatment (one or two injections), ipsilesional hearing was impaired in 32/34 patients (94%) at 0.5 Hz (mean = 50 dB HL, range = 10–120), 1 Hz (mean = 68 dB HL, range = 10–120), 2 Hz (mean = 82 dB HL, range = 25–120), and 4 Hz (mean = 86 dB HL, range = 40–120), with an increased average CPT-AMA hearing loss of 73 ± 24%. Note that no PTA was available after the first gentamicin treatment in 5 out 8 patients who received two gentamicin injections.

## Discussion

Vestibular pre-habilitation by use of intratympanic gentamicin prior to VS resection allows for a stepwise reduction in peripheral-vestibular function. In our study, all 34 patients showed significant reductions in aVOR gain and increases in CSA for all three SCCs compared to baseline. Changes were most profound for the horizontal SCC, and the number of patients showing normal function remained significantly larger for the anterior SCC compared to the horizontal and posterior canal. Thus, the data confirms our hypothesis of preferential damage to selected SCCs due to intratympanic gentamicin application with relative sparing of the anterior canal in VS patients. Surprisingly, we also noted relative sparing of ipsilesional anterior-canal function at baseline.

### The Impact of Intratympanic Gentamicin on Peripheral-Vestibular Function in VS

The application of intratympanic gentamicin resulted in a substantial reduction in peripheral-vestibular function as assessed both by an overall rater-dependent score and by aVOR gains and CSA. Hearing loss was already substantial at baseline in most patients, remaining almost stable after gentamicin treatment. This supports the concept of pre-habilitation and emphasizes the effectiveness of gentamicin as a predominantly vestibulotoxic substance. Noteworthily, relative sparing of anterior-canal function was preserved after gentamicin application, speaking against the hypothesis that this vestibulotoxic substance eliminates any residual vestibular function in an unselective manner. In contrast, our data suggests that the anterior SCC is less susceptible to gentamicin toxicity. We have previously observed a similar effect in patients with bilateral-vestibular loss (22) due to systemic aminoglycoside treatment, and our current data confirms this characteristic pattern of anterior-canal sparing. Whereas, the manner of application (intravenous vs. intratympanic) seems to have little effect on the pattern, the underlying cause for relative sparing of anterior-canal function after aminoglycoside treatment remains unclear.

Currently, the pathophysiological mechanisms for anterior-canal sparing after aminoglycoside exposure remain unresolved. Potential explanations for anterior canal sparing [as previously discussed by (22)] after gentamicin instillation include an accumulation of gentamicin in the most basal parts of the vestibular organ, following the pull of gravity. Thus, the posterior and horizontal canal—being located below the anterior canal—would be exposed to higher doses of gentamicin. Alternatively, either the anterior SCC could be less vulnerable to gentamicin or it recovers more quickly after gentamicin-induced damage. Theoretically, anterior canal sparing in vHIT could be a measurement bias. This, however, seems unlikely for several reasons. First, sparing was restricted to certain disorders as reported 2016 by our group (22). Second, the vertical SCCs are always tested in pairs according to their planes of stimulation and head impulses of similar velocities were applied for all vertical SCCs. Third, as previously reported anecdotally, patients reported oscillopsia for upward head movements only, but not for downward head movements, matching their vHIT pattern of spared anterior SCCs (22).

Observed decreases in peripheral-vestibular function after intratympanic gentamicin ranged between 16% (anterior canal), 26% (posterior canal), and 32% (horizontal canal) as assessed by the aVOR and between 95% (horizontal canal), 174% (posterior canal), and 195% (anterior canal) reflected by increased CSA. In comparison, for the anterior canal the reduction in aVOR gain was smaller (showing a statistical trend) and the increase in CSA was significantly larger compared to the horizontal SCC. The effect size of gentamicin application depended on the initial aVOR gain, being larger in those patients with relatively preserved aVOR function. Thus, with aVOR function relatively spared at baseline, anterior-canal function may experience a steeper decrease than horizontal-canal function, which already at baseline was more profoundly impaired. Nonetheless, aVOR-gain changes were larger for the horizontal canal than for the anterior canal, underlining the relative sparing of anterior-canal function.

Previous studies assessed aVOR reduction after intratympanic gentamicin treatment in VS by bedside head-impulse testing (13, 14, 33) and caloric irrigation (6, 7, 13, 14, 20, 33, 34). None of the published studies reported aVOR gains and/or CSA of all six SCCs assessed by vHIT.

From the perspective of pre-habilitation, residual anterior-canal function despite gentamicin treatment may make VS patients more prone to symptoms related to sudden loss of anterior-canal function after VS resection—i.e., predominantly vertical spinning vertigo. Also, a first dosage of gentamicin was ineffective in 8/34 patients, requiring a second dose. This emphasizes the need to monitor the effect of intratympanic gentamicin application by vHIT of all six SCCs.

#### Effect of Gentamicin Instillation on the aVOR in Menière's Disease

To further elaborate on the impact of intratympanic gentamicin treatment, we compared our results to published treatment studies on MD. Previously, others compared aVOR gains before and after gentamicin instillation in 32 patients with unilateral MD, reporting an average of 40% decrease in aVOR gain for all three canals (35). The observed drop in aVOR gain of 0.24–0.35 was similar for all three SCCs, showing no anterior-canal sparing. However, in this study only aVOR-gain values were assessed and no overall rating of the vHIT response was provided. This is distinct from our approach, possibly explaining the discrepant findings. In another study, average gains after treatment in 31 patients with unilateral definite MD were 0.61, 0.69, and 0.47, respectively, for the anterior, horizontal, and posterior SCC (36). Corresponding rates of reduction of vestibular function were 48, 26, and 36%, respectively. Using magnetic search coils to assess aVOR gains before/after gentamicin instillation in patients with MD, others reported average (±1SD) aVOR gains after intratympanic gentamicin of 0.40 ± 0.12, 0.35 ± 0.14, and 0.31 ± 0.14, for the horizontal, anterior, and posterior ipsilesional SCC (37). In another study from the same group, 18 patients with unilateral MD were followed up after a single or multiple gentamicin instillations after 12 months, again using magnetic search coils. Resulting aVOR gains after intratympanic gentamicin were 0.53 ± 0.27 (Δgain = 0.32 ± 0.35), 0.47 ± 0.16 (Δgain = 0.31 ± 0.24), and 0.43 ± 0.21 (Δgain = 0.32 ± 0.27) for the horizontal, anterior, and posterior ipsilesional SCC (38). Thus, in these studies on the effect of gentamicin on the different SCCs in MD, no relative sparing of anterior-canal function was observed, contrasting our VS data. Potential explanations are differences in the analysis (we considered increased CSA as indicative for peripheral-vestibular loss also) and the recording system (vHIT vs. magnetic search coils).

In another study focusing on horizontal aVOR gains before and after gentamicin instillation in 20 patients with unilateral MD, both a delayed effect of gentamicin instillation with maximal aVOR gain reduction observed after 1 month and partial recovery after 3 months post-instillation were observed (39). Thus, timing after gentamicin instillation seems to be important; specifically, too early post-instillation vHIT assessment may underestimate the effect of gentamicin instillation, and in case of delayed (i.e., after more than 2 months) surgical removal of the VS the benefit of the pre-habilitation treatment may be reduced.

### Anterior-Canal Sparing in VS at Baseline

In our study, baseline vHIT measurements demonstrated significantly more often ipsilesional sparing of anterior-canal function than horizontal or posterior canal function. For the anterior canal, ipsilesional aVOR gains were not significantly reduced and ipsilesional CSA were not significantly increased compared to the healthy side. In a recent study from our laboratory on the characterization of unilateral peripheral-vestibular deficits in a mixed cohort [using vHIT and ocular/cervical vestibular-evoked myogenic potentials (VEMPs)], the VS subgroup (*n* = 55) presented with anterior-canal sparing as well (25).

Noteworthily, horizontal head impulses are usually applied with higher peak head velocities than vertical head impulses and thus, may be more sensitive in detecting SCC loss of function. Whereas, in relation to horizontal canal function, this may explain relative sparing of anterior-canal function, this is not the case when comparing vertical head impulses (i.e., anterior vs. posterior canal function).

Previously, such anterior-canal sparing has been reported by others using the same vHIT goggles (40, 41). Specifically, the fraction of VS patients (*n* = 41) with loss of function of the ipsilesional anterior SCC was significantly lower than that of the horizontal (9 vs. 28, *p* < 0.001, Bonferroni-corrected for multiple tests) and the posterior (9 vs. 20, *p* = 0.040) SCC in one study (41). Likewise, in another study, rates of impairment of the ipsilesional horizontal, anterior, and posterior SCC were 34/55 (62%), 20/55 (36%), and 31/55 (57%), with significantly lower rates for the anterior canal than for the horizontal (*p* = 0.002, McNemar-test) and posterior (*p* = 0.031) SCC (40).

The reason for this anterior-canal sparing in untreated VS patients remains unclear. With the mechanism of damage being tumor growth and compression, one may speculate that those nerve fibers originating from the anterior SCC are either less prone to compression or are better protected, e.g., by a more remote location to the origin of the tumor growth.

### Correlation of Tumor Size and Vestibular Loss

In our study with VS of varying size (range = 2–50 mm), we found that the size of the VS had a significant impact on the extent of peripheral-vestibular loss of function. Specifically, ipsilesional vHIT gains at baseline showed a significant inverse correlation with the VS diameter. Previously, others have reported similar findings (40). Specifically, using an audio-vestibular test battery (vHIT, PTA, ocular/cervical VEMPs), damage to at least one vestibular sensor was less frequent in those patients with a VS diameter of ≤ 14 *mm* compared to those with a VS diameter >14 mm (39 vs. 100%). Likewise, a significant association between tumor size and horizontal-SCC function (assessed by vHIT or caloric irrigation) was reported by several groups (42–44). Noteworthily, others have failed to show such a relationship (41, 45). This discrepancy may be related to the patient sample size, the parameters chosen for comparison, and the statistical analyses performed.

### Limitations

Our study has several limitations. This includes the individually varying doses of gentamicin applied intratympanically and also the lack of a control group receiving placebo instead. Furthermore, we did not assess the impact of gentamicin instillation on utricular and saccular function and did not assess the symptom severity after VS resection. Thus, based on the study design applied here (focusing on aVOR properties before and after gentamicin instillation), we cannot make any conclusions about the impact of vestibular pre-habilitation using intratympanic gentamicin application on recovery after VS resection. To answer this clinically important question, further research and specifically designed studies will be needed, comparing the outcome after VS resection in different treatment groups (pre-habilitation vs. standard treatment only).

## Conclusions

Intratympanic gentamicin application resulted in a substantial reduction of peripheral-vestibular function in all three SCCs. Relative sparing of anterior-canal function noted at baseline was preserved after gentamicin treatment, with a significantly larger decrease in peripheral-vestibular function in the horizontal SCC compared to the anterior SCC (as reflected by changes in CSA). This has two major implications. First, our data confirms that pre-surgical intratympanic gentamicin was successful in reducing residual peripheral-vestibular function before surgery, thus further supporting the concept of vestibular pre-habilitation. Second, relative sparing of anterior-canal function in VS patients at baseline and after gentamicin application suggests that the vulnerability of the distinct SCCs to both local damage of nerve fibers due to tumor growth and to vestibulotoxic substances varies. The reasons for such relative sparing of the anterior SCC remain to be determined.

## Data Availability Statement

The raw data supporting the conclusions of this article will be made available by the authors, without undue reservation.

## Ethics Statement

The studies involving human participants were reviewed and approved by Cantonal Ethics Committee Zurich. The patients/participants provided their written informed consent to participate in this study.

## Author Contributions

AT conception and design of the experiments, analysis and interpretation of data, drafting and revision of the article critically for important intellectual content. CB analysis and interpretation of data and revision of the article critically for important intellectual content. EB data collection and revision of the article critically for important intellectual content. AH, VW, and KW conception and design of the experiments, interpretation of data, revision of the article critically for important intellectual content. All authors have approved the final version of the manuscript, all persons designated as authors qualify for authorship, and all those who qualify for authorship are listed.

## Conflict of Interest

KW was supported by the Swiss National Science Foundation (320030_166346) and the Uniscientia Stiftung, Vaduz, Liechtenstein. He acts as an unpaid consultant and has received funding for travel from Otometrics. The remaining authors declare that the research was conducted in the absence of any commercial or financial relationships that could be construed as a potential conflict of interest.
